# Clinical Advantages and Pitfalls of Pulsed Field Ablation: How to Prevent ALH Syndrome

**DOI:** 10.1002/joa3.70482

**Published:** 2026-10-07

**Authors:** Hiroshi Nakagawa, Quim Castellvi, Masafumi Sugawara, Atsushi Ikeda, Salman Farshchi‐Heydari, Jennifer Maffre, Ayman A. Hussein, Pasquale Santangeli, Walid I. Saliba, Yukihito Higashi, Warren M. Jackman

**Affiliations:** ^1^ Department of Cardiovascular Medicine Cleveland Clinic Cleveland Ohio USA; ^2^ Department of Engineering Universitat Pompeu Fabra Barcelona Spain; ^3^ Department of Cardiology Chiba University Chiba Japan; ^4^ Department of Cardiology Akiru Municipal Medical Center Tokyo Japan; ^5^ Johnson & Johnson MedTech Irvine California USA; ^6^ Department of Regenerative Medicine, Research Institute for Radiation Biology and Medicine Hiroshima University Hiroshima Japan; ^7^ Heart Rhythm Institute University of Oklahoma Oklahoma City Oklahoma USA

**Keywords:** catheter ablation, electrophysiology mapping, tachyarrhythmias

## Abstract

Catheter ablation for tachyarrhythmias was first introduced in the early 1980s using direct‐current (DC) energy. Following the clinical introduction of radiofrequency (RF) ablation in the late 1980s, thermal ablation technologies became the foundation of catheter ablation because of their superior controllability, efficacy, and safety. In 2014, Wittkampf developed a DC‐energy‐delivery catheter system preventing electrical arcing, thereby initiating a modern “revival of DC‐ablation” based on catheter ablation using electroporation. Subsequent development of high‐voltage, short‐duration pulse‐train delivery led to the emergence of pulsed field ablation (PFA). Exposure of myocytes to high‐voltage electric fields induces reversible electroporation through nanoscale pore formation within the lipid bilayer, whereas higher electric‐field delivery produces irreversible electroporation, resulting in permanent membrane disruption, apoptosis, and durable lesion formation.

PFA is regarded as a predominantly nonthermal and potentially tissue‐selective ablation modality. Nevertheless, clinical trials of pulmonary vein isolation for atrial fibrillation have demonstrated that PFA is noninferior to conventional thermal ablation strategies, although clear superiority—other than shorter procedural times—has not yet been established. Importantly, recent studies suggest that electrode–tissue contact remains a critical determinant of durable lesion formation. Accumulating evidence indicates that PFA is associated with new complications not commonly observed with thermal ablation, including acute kidney injury, stroke, coronary vasospasm, myocardial ischemia, ventricular fibrillation, and sudden cardiac death. Although the mechanisms remain incompletely understood, hemolysis‐related injury induced by high‐voltage pulse delivery may represent an important contributing mechanism. Development of PFA systems capable of real‐time prediction of irreversible lesion size may be essential for optimizing both procedural efficacy and safety.

## Graphical abstract

Pulsed‐field (PF) Ablation Index (combined with average contact force and number of PF pulses) accurately predicted lesion depth in the prospective validation study. Importantly, no lesion formation was observed in the absence of electrode–tissue contact when the ablation electrode was positioned only 1–2 mm away from the endocardium.

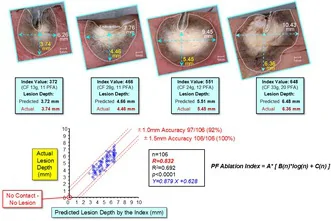

## 1 Introduction

Catheter ablation for tachyarrhythmias, introduced in the early 1980s, initially relied on direct‐current (DC) energy delivered using external defibrillators. During the following three decades, radiofrequency (RF) energy and cryothermal ablation became the dominant energy sources for catheter‐based arrhythmia therapy. In the 2020s, pulsed field ablation (PFA) emerged as a novel nonthermal ablation modality and has entered clinical practice for pulmonary vein (PV) isolation in patients with atrial fibrillation (AF).

This review summarizes the historical development of PFA, the mechanisms of lesion formation, and the most recent clinical experience, with particular attention to the current limitations and unresolved challenges of this rapidly evolving technology.

### 1.1 Historical Development of Pulsed Field Ablation

The origins of PFA can be traced back to observations reported in 1979. Fontaine and colleagues in Paris reported complete atrioventricular (AV) block immediately after external defibrillation during a clinical electrophysiology study [1]. They postulated that the DC delivered during external defibrillation may have inadvertently been transmitted to the His bundle recording catheter, resulting in injury to the AV conduction system.

Subsequently, Gonzalez and colleagues performed experimental studies of DC ablation in 10 mongrel dogs [2]. A 6‐Fr quadripolar catheter (1‐mm electrode length) was positioned at the site of recording His bundle potentials, and DC shocks (35 J, 1–2 shocks) were delivered between the catheter electrode and an external patch. Complete AV block was produced in all animals and persisted for 3 months in 9 of the 10 dogs.

In 1982, Scheinman and colleagues reported the first clinical application of DC ablation for drug‐refractory supraventricular tachycardia [3]. Five patients with structural heart disease complicated by syncope or heart failure underwent DC ablation with the intentional creation of complete AV block. Using a custom‐made quadripolar catheter, two 500‐J shocks were typically delivered at the site exhibiting the largest His bundle potential, successfully producing complete AV block in all patients. During follow‐up, one patient experienced sudden cardiac death 6 weeks after ablation, presumably due to a cardiac arrhythmia. In the remaining four patients, complete AV block persisted for 4–12 months of follow‐up, and permanent pacemaker implantation resulted in marked symptomatic improvement and increased exercise tolerance.

Around the same time, Gallagher and colleagues also reported the results of DC ablation in nine patients with drug‐refractory supraventricular tachycardia [4]. A 6‐Fr tripolar catheter was positioned at the His bundle recording site, and DC shocks of 200–300 J were delivered between the catheter electrode and an external patch using an external defibrillator. Complete AV block was achieved in 8 of 9 patients, whereas AV nodal modification was observed in the remaining patient. During the 2–6‐month follow‐up, no arrhythmia recurrence or major complications were observed. These two landmark reports marked the beginning of the era of catheter ablation therapy.

Subsequent experimental studies of DC ablation in ventricular myocardium using canine models demonstrated that ablation lesions were characterized histologically by contraction band necrosis and intramyocardial hemorrhage [5, 6]. In addition, the occurrence of ventricular arrhythmias following DC energy delivery suggested a potential proarrhythmic effect of this ablation technique [3, 5].

In 1986, the DC Ablation Registry reported outcomes in 88 patients with ventricular tachycardia (VT), including post–myocardial infarction VT (63 patients), cardiomyopathy (16 patients), arrhythmogenic right ventricular cardiomyopathy (10 patients), valvular heart disease (6 patients), idiopathic VT (6 patients), and other etiologies (6 patients) [7]. In this registry, 2 DC shocks of approximately 200 J were typically delivered. During a follow‐up period of 10 ± 8 months, outcomes were categorized as asymptomatic without antiarrhythmic drugs in 33% of the patients, asymptomatic with antiarrhythmic drugs in 38%, and unsuccessful in 20%. However, the mortality rate during follow‐up was substantial (22 of 88 patients, 25%), including 4 deaths within 24 h (3 due to severe hypotension with electromechanical dissociation and 1 during induction of anesthesia), 12 sudden cardiac deaths (VT/VF), 4 deaths from severe heart failure, and 2 non‐cardiac deaths.

In an effort to reduce complications associated with DC ablation—including cardiac tamponade, heart failure, pro‐arrhythmia, and sudden death—low‐energy DC ablation (15–30 J, 4–6 shocks) was subsequently applied in 60 patients with accessory pathways, achieving successful ablation of the accessory pathways without complications in 55 patients (92%) [8]. During this period, several strategies were investigated to mitigate complications associated with DC ablation, including: (1) reduction of delivered DC energy; (2) increasing the surface area of the ablation electrode; (3) using cathodal polarity discharge to reduce electrical arcing; and (4) delivering DC energy as short‐time constant capacitive discharges (60–100 μs) in pulse trains to prevent arcing [9].

The clinical introduction of RF energy for ablation in the late 1980s showed improved safety and controllability, and rapidly led to the abandonment of DC ablation [10, 11, 12]. For more than three decades thereafter, RF energy remained the dominant modality for catheter ablation therapy [10, 11, 12, 13]. Major complications of RF ablation include PV stenosis, phrenic nerve injury, esophageal injury, and steam pop‐related cardiac tamponade. Importantly, extensive clinical experience and technological refinement have markedly reduced the incidence of these complications through strategies such as avoiding RF delivery within the PVs, using low‐energy applications near vulnerable extracardiac structures, and controlling lesion depth with Ablation Index–guided ablation [14, 15]. In parallel, efforts to shorten procedural duration have driven the development of single‐shot ablation technologies, including cryo‐balloon catheters [13], with PFA representing the latest evolution of this concept.

Notably, the concept of DC‐based ablation, which had largely disappeared by the early 1990s, has re‐emerged 30–40 years later as PFA, which uses lower‐energy DC and/or more than 1000 times shorter range pulses delivered in rapid pulse trains [16, 17, 18].

### 1.2 Mechanisms of Reversible and Irreversible Lesion Formation With Pulsed Field Ablation

In 2014, Wittkampf and colleagues reported experimental studies using a swine model in which a circular catheter (7 Fr, 12‐mm diameter with eight 2‐mm electrodes) was positioned within the epicardial space [18]. DC energy below the threshold for electrical arcing was delivered as a single cathodal 200‐J shock between all 8 electrodes and an external patch. Histopathological examination 3 months later demonstrated well‐defined fibrotic ventricular ablation lesions with a mean depth of 6.4 ± 2.6 mm (range, 0–14 mm). They described this approach as “catheter ablation using electroporation,” which may be viewed as a technological re‐emergence of the original concept of DC ablation more than three decades after its initial development, “Revival of DC Ablation” [17, 18].

The cardiomyocyte membrane is composed of an electrically insulated lipid bilayer that regulates excitation, conduction, and contraction through the coordinated opening and closing of complex ionic channels. Exposure of myocardial cells to high‐voltage, short‐duration electric fields can transiently increase membrane permeability by creating nanoscale pores within the lipid bilayer, a process termed “electroporation.” This phenomenon has been widely used since the 1990s to introduce large molecules, including genetic material, into cells for gene transfer applications [19]. When relatively low electric energy is delivered, the cell membrane reseals through intrinsic repair mechanisms, allowing normal cellular function to recover. This is so‐called “***reversible electroporation***” [19]. However, when the electric field strength and pulse duration exceed a critical threshold, “***irreversible electroporation***” occurs, resulting in extended membrane disruption, apoptosis, and cell death, thereby producing durable ablation lesions (Figure 1) [20].

**FIGURE 1:**
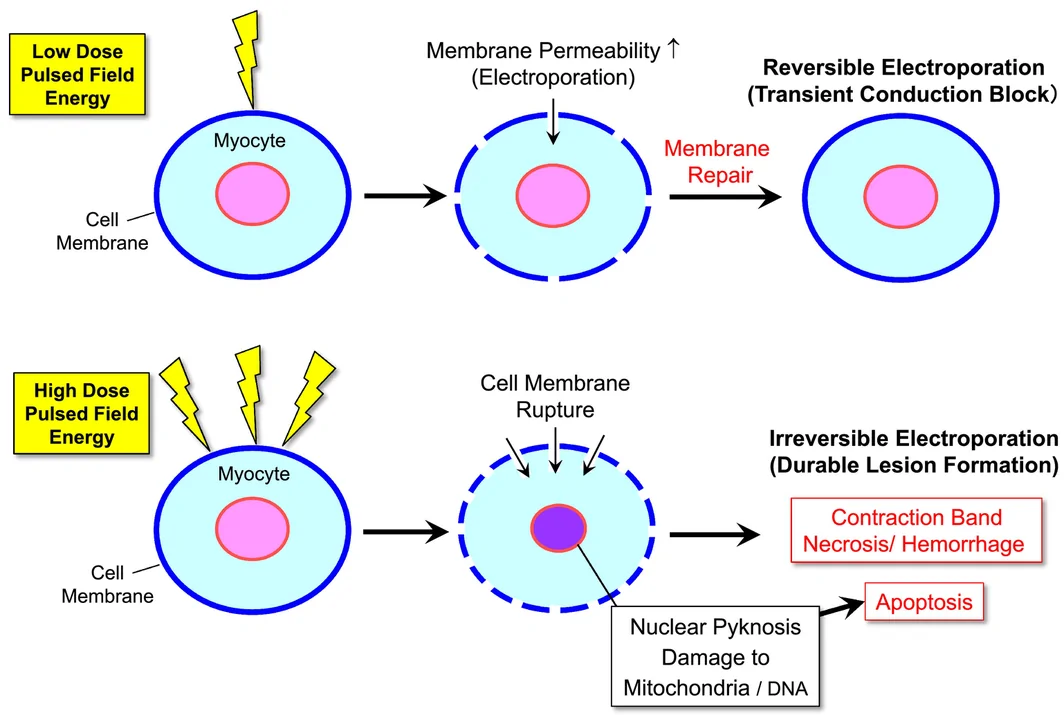
Mechanisms of reversible and irreversible electroporation during pulsed field ablation. Schematic illustration of the cellular mechanisms underlying reversible and irreversible electroporation induced by pulsed field ablation (PFA). See the text for details.

In experimental studies using a closed‐chest swine model, we evaluated lesion formation using a current‐controlled PFA generator (monopolar, biphasic microsecond pulses; CENTAURI, CardioFocus Inc.) delivered between a 7 Fr, 4 mm tip electrode with a contact force (CF) sensor (TactiCath SE, Abbott Inc.) and an external patch electrode [21]. PFA applications delivered within the left ventricle (35 Amperes, total duration of 1.6 ms) produced effective PFA lesions. Lesion depth increased significantly with increasing electrode–tissue CF (5–55 g), ranging from approximately 2 to 8 mm. In contrast, when the catheter electrode was positioned 1–2 mm away from the endocardial surface without contact, no lesion formation was observed (Figure 2), highlighting the critical role of electrode–tissue contact in PFA lesion formation.

**FIGURE 2:**
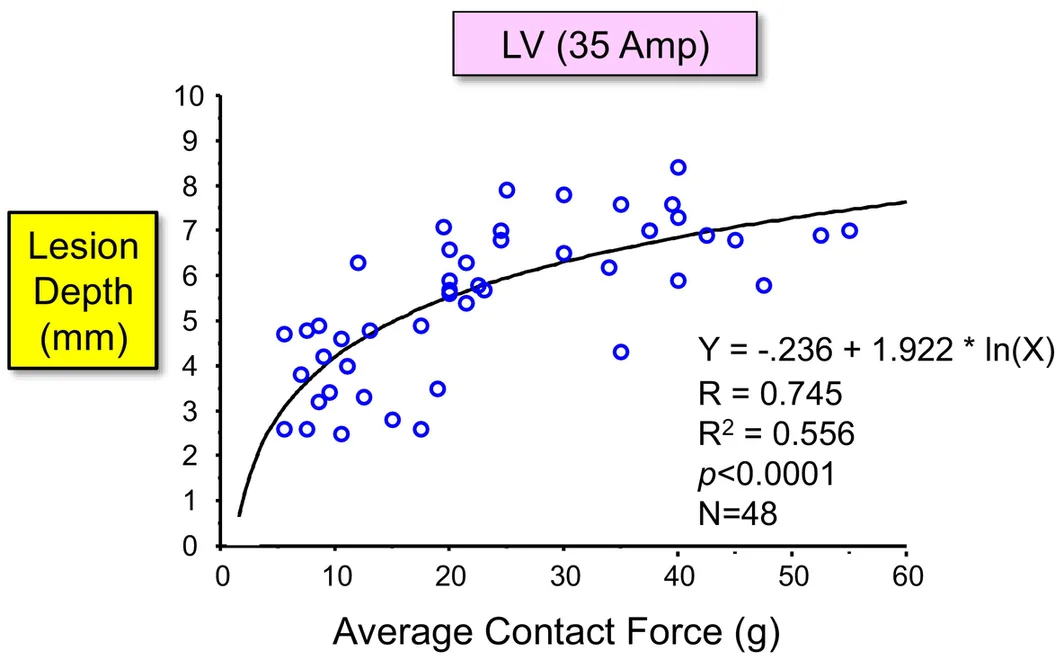
Relationship between catheter contact force and lesion depth during pulsed field ablation. Correlation between catheter–tissue contact force and lesion depth during PFA delivery. From Nakagawa et al. [21] with permission.

After a minimum of 2 h post‐PFA, the ablation lesions were well demarcated using triphenyl tetrazolium chloride (TTC) staining and demonstrated three distinct zones: (1) dark central zone; (2) pale zone; and (3) hyperstained red zone (Figure 3) [21]. The microscopic histological examination using hematoxylin and eosin (H&E) staining of the dark central zone exhibited destruction of myocyte architecture with contraction band necrosis and nuclear pyknosis, consistent with an acute necrosis zone. The presence of contraction band necrosis suggests significantly increased Ca + influx into the myocyte associated with cell membrane rupture, which has been shown to occur in the setting of reperfusion injury in acute myocardial infarction [22]. Red blood cell infiltrations and increased interstitial space indicate hemorrhage and edema resulting from increased microvascular permeability induced by PFA [23]. Importantly, there is no evidence of coagulation necrosis resulting from thermal injury, which is commonly observed in RF ablation lesions [14, 24].

**FIGURE 3:**
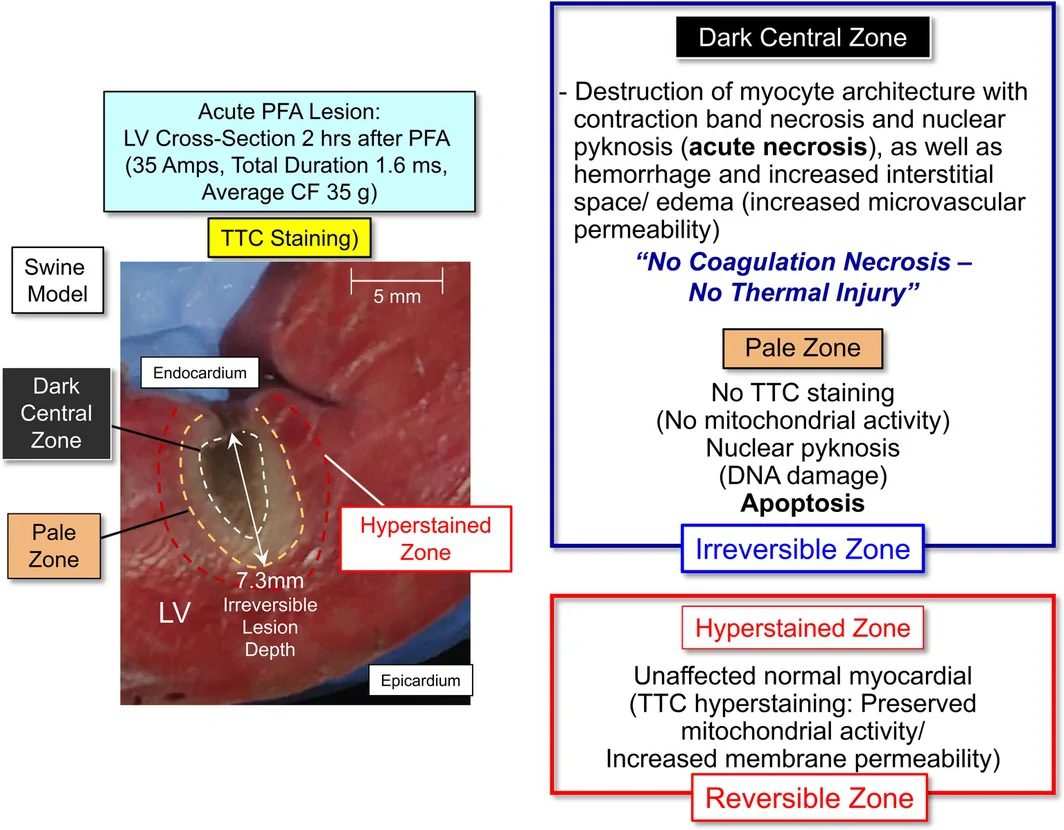
Histopathological characteristics of lesion formation following pulsed field ablation. Representative histopathological features and spatial distribution of myocardial injury following PFA. See the text for details.

Histology (H&E staining) of the pale zone with TTC staining exhibited relatively preserved myocyte architecture with nuclear pyknosis. No TTC staining suggests damage to the mitochondria (no activity of succinate dehydrogenase), and nuclear pyknosis indicates damage to DNA [25]. These histochemical observations are consistent with an apoptotic pathway [25]. At the outermost layer, a TTC‐hyperstained red zone was observed. Histology of this zone showed normal cardiomyocyte morphology. Positive staining of TTC indicates preserved mitochondrial activity, suggesting a reversibly affected area [25, 26, 27]. Jeong et al. utilized TTC staining to delineate reversible and irreversible PFA lesion boundaries in a potato model. Similar to the PFA myocardial lesions, they found that: (1) a TTC‐unstained pale area delineated the irreversible lesion boundary; and (2) a TTC‐hyperstained deep‐red area was formed surrounding the irreversible lesion boundary, indicating the reversibly affected area [28]. The TTC‐hyperstained deep‐red area was located peripherally at lower PFA voltage delivery sites (100–200 V/cm), while the TTC‐unstained area was located centrally at higher PFA voltage delivery sites (> 250 V/cm) [28]. TTC is consumed by succinate dehydrogenase in the mitochondria, resulting in a dark‐red color staining of the cell. The color intensity of TTC staining could be related to the degree of mitochondrial activity. Several experimental studies have shown that PFA delivery results in ATP leakage, and preserved or enhanced mitochondrial activity compensates for the ATP depletion over 8 to 24 h after PFA application [29]. consistent with reversible injury.

COX and SDH dual staining (COX/SDH) have been developed for quantitative analysis of mitochondrial activity, staining cells with normal mitochondrial function in light brown [30]. Consistent with TTC staining, COX/SDH staining of the PFA lesion showed no staining of the central zone surrounded by a hyperstained dark brown zone, delineating irreversible and reversible lesion boundaries, respectively [28].

Notably, this relatively broad outer zone of reversible injury appears to represent a unique characteristic of PFA lesions that is not observed with conventional thermal RF ablation. These findings suggest that PFA creates a central core of irreversible myocardial injury surrounded by a substantially larger region of reversible electroporation capable of producing prolonged transient conduction block beyond the period of PFA energy delivery (Figures 3 and 4).

**FIGURE 4:**
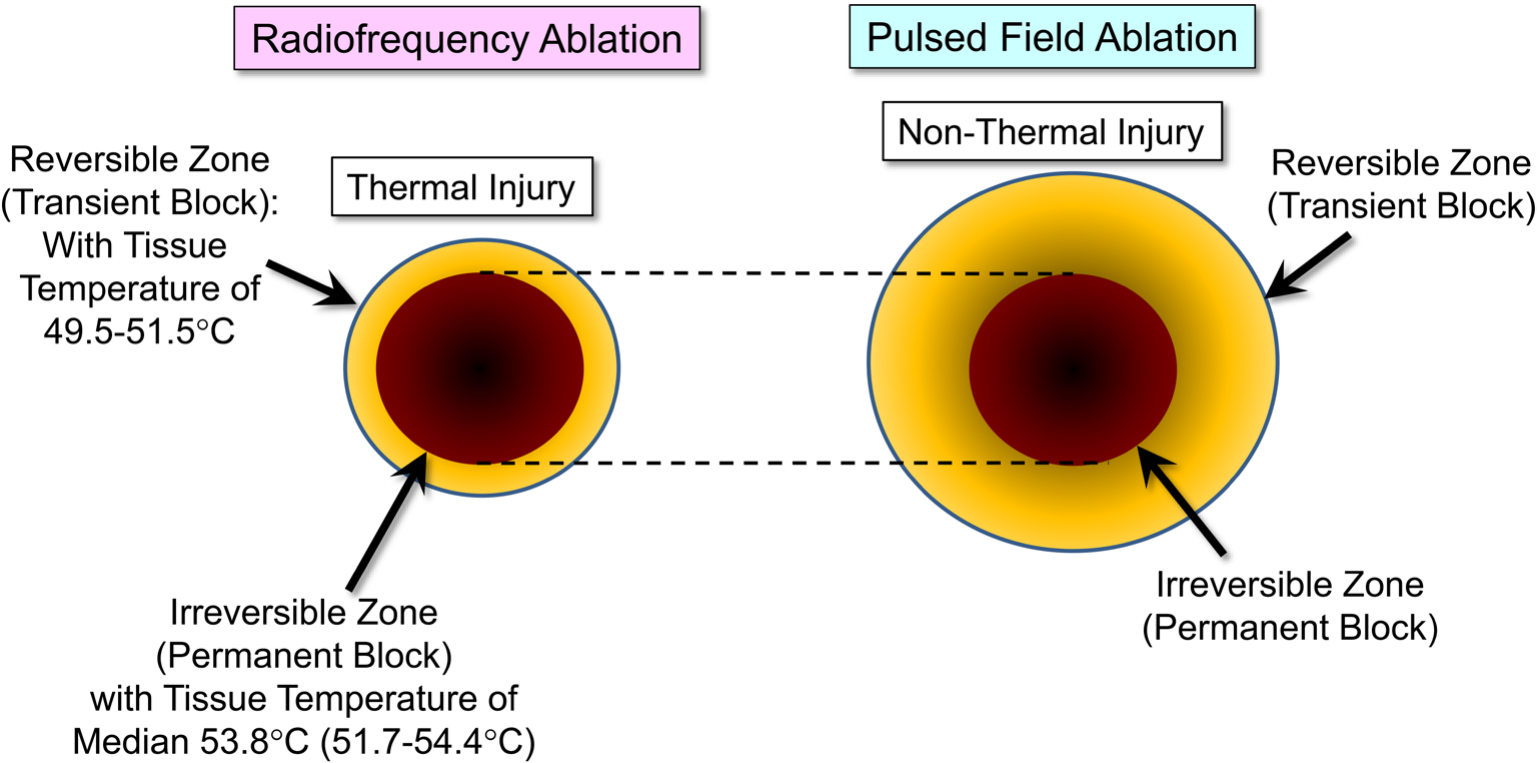
Comparison of reversible and irreversible lesion distribution between pulsed field ablation and radiofrequency ablation. Schematic comparison of the spatial extent of reversible and irreversible myocardial injury produced by pulsed field ablation and conventional radiofrequency ablation.

In a swine model, we examined the relationship between changes in intracardiac electrograms recorded from a 7.5Fr ablation catheter with a 3.5‐mm tip electrode before and after PFA delivery and the size of irreversible PFA lesions [31]. The following electrogram parameters were analyzed before and after PFA: (1) bipolar ventricular potential amplitude (peak‐to‐peak of the RS segment); (2) unipolar ventricular potential amplitude (peak‐to‐peak of the RS segment) recorded from the distal and second electrodes; (3) distal unipolar potential mean negative *dV/dt* (defined as the amplitude‐to‐duration ratio of the downstroke); and (4) unipolar ST‐segment amplitude (injury current) recorded from the distal and second electrodes. These electrogram parameters demonstrated no or poor correlation with PFA lesion size (Figure 5) [31]. This likely reflects the presence of a relatively broad zone of transient conduction block associated with reversible electroporation, which may obscure the relationship between acute electrogram attenuation and the extent of irreversible myocardial injury. Accordingly, the degree of electrogram reduction or disappearance is *not* a reliable predictor of PFA lesion size, underscoring the importance of non‐electrogram‐based quantitative approaches, such as an ablation index using parameters of CF and number of PFA pulses, for real‐time prediction of durable lesion formation [31, 32].

**FIGURE 5:**
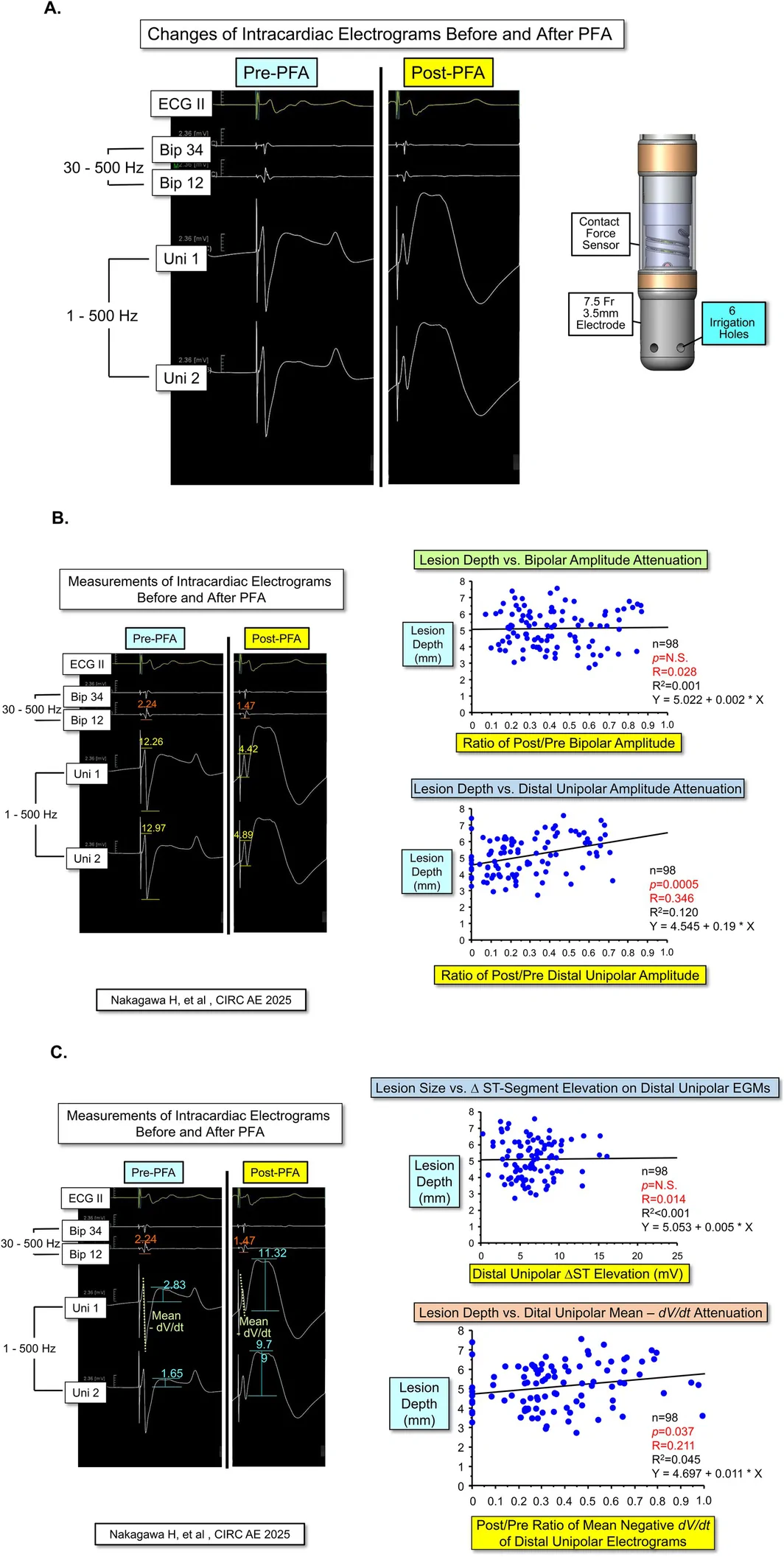
Relationship between attenuation of intracardiac electrograms after PFA and lesion size. (A) The peak‐to‐peak amplitude of bipolar electrograms and unipolar electrograms (recorded from the distal and second electrodes) and the mean negative *dV/dt* of the downstroke of the distal unipolar electrograms significantly decreased after PFA. (B) No significant relationship existed between the ratio of post/pre‐PFA of the bipolar electrogram amplitude and lesion depth (*R* = 0.028, *p* = N.S.). A poor relationship existed between the ratio of post/pre‐PFA of the distal unipolar electrogram amplitude and lesion depth (*R* = 0.346). (C) No significant relationship existed between the delta increase of ST‐segment amplitude of the distal unipolar electrogram and lesion depth (*R* = 0.014, *p* = N.S.). A poor relationship existed between the ratio of post/pre‐PFA of mean negative *dV/dt* of the downstroke in the distal unipolar electrogram and lesion depth (*R* = 0.211, *p* = 0.037).

### 1.3 Clinical Outcomes and Current Limitations of Pulsed Field Ablation for Pulmonary Vein Isolation

Since the early 2020s, clinical trials evaluating PFA for PV isolation in patients with AF have been initiated using multi‐electrode circular or basket‐shaped catheters (typically 9–20 electrodes).

One such system employs a 9‐Fr circular catheter with nine 3‐mm electrodes and a 25‐mm diameter (PulseSelect, Medtronic Inc.). Using biphasic bipolar pulse trains (1400–1500 V, total pulse duration 100–200 ms, with each application consisting of four burst trains), PV isolation was performed in patients with AF. In the PULSED‐AF trial, 150 patients with paroxysmal AF and 150 patients with persistent AF underwent ablation, with an average of 48 ± 15 and 57 ± 20 PFA applications, respectively, achieving acute PV isolation in all 300 patients. At 12‐month follow‐up, freedom from atrial arrhythmia recurrence was 69.5% in patients with paroxysmal AF and 62.3% in patients with persistent AF. Reported complications included 1 stroke, MRI‐detected silent cerebral lesions in 4 of 45 patients (9%), cardiac tamponade in 1 patient, and 2 deaths [33].

In a clinical study of 186 patients with paroxysmal AF, PV isolation was performed using a variable‐loop circular catheter (8.5‐Fr shaft; 10 electrodes, each 3 mm in length; irrigation rate 4 mL/min; VARIPULSE, Johnson & Johnson MedTech Inc.) in combination with a multichannel PFA generator delivering bipolar, microsecond biphasic pulses (1800 V; total pulse train duration 239 ms, repeated three times at 10‐s intervals). At least 12 PFA applications per PV were delivered, with a mean procedure time of 70.1 ± 27.7 min and a PFA delivery time of 26.7 ± 14.0 min. Acute entrance block was achieved in all pulmonary veins. At 12‐month follow‐up, 70.9% of patients remained free from atrial arrhythmia recurrence. No primary adverse events were observed, including either atrio‐esophageal fistula, PV stenosis, cardiac tamponade, phrenic nerve injury, stroke or other cerebrovascular events, thromboembolism, transient ischemic attack, myocardial infarction, pericarditis, major vascular access complications, or device‐ or procedure‐related death [34].

Another widely studied system utilizes a 12 Fr, 20‐electrode catheter (FARAWAVE, Farapulse, Boston Scientific Inc.), which can be configured as a basket (31 mm diameter) or a flower (35 mm diameter). This system delivers biphasic bipolar pulse trains (1800–2000 V, 200 ms duration), with each application consisting of five burst trains. In a clinical trial involving 305 patients with paroxysmal AF, PV isolation was achieved in 99.6% of patients, typically requiring ≥ 8 applications per PV. At 12‐month follow‐up, 73.1% of patients remained free from atrial arrhythmia recurrence, compared with 71.3% in a control cohort of 302 patients treated with conventional thermal ablation systems (RF ablation in 167 patients and cryo‐balloon ablation in 135 patients). Reported complications in the PFA group included 1 death, 1 transient ischemic attack, 2 cases of cardiac tamponade, 1 case of pulmonary edema, and 1 case of pericarditis [35].

In a retrospective analysis of 1184 patients (62% with paroxysmal AF) undergoing PV isolation with the Farapulse system across seven European centers, complete elimination of PV potentials was achieved in all patients during the index procedure. However, during a median follow‐up of 5 months, at least 272 patients (23%) experienced recurrence of atrial arrhythmias. Among the 144 patients who underwent repeat procedures, PV reconnection was observed in 62% of patients. Importantly, there was marked inter‐center variability in the incidence of durable PV isolation, ranging from 9% to 64% of patients across the seven centers. These findings highlight the limitations of using acute PV potential elimination as a procedural endpoint, indicating the need to standardize PFA‐based PV isolation techniques to improve lesion durability and clinical outcomes [36].

Taken together, these clinical trials indicate that PFA is noninferior to conventional thermal ablation strategies (RF or cryo‐balloon ablation) for AF ablation, although clear superiority has not yet been demonstrated, except for shorter procedure times [37, 38].

### 1.4 Importance of Electrode–Tissue Contact for Durable PFA Lesion Formation

PFA is frequently described as a primarily “nonthermal” energy modality that may be less dependent on catheter contact than thermal ablation. However, recent experimental studies have shown that electrode–tissue contact remains a critical determinant of effective lesion formation [21, 31, 32, 39]. Delivery of electric current into myocardial cells is highly sensitive to the distance between the electrode and the tissue. Even small separations between the electrode and the endocardial surface can markedly reduce the electric field intensity within the target myocardium, potentially preventing the threshold required for “irreversible” electroporation from being reached.

In experimental studies using a swine beating‐heart model, ventricular ablation was performed with a 7.5 Fr, 3.5 mm tip‐electrode focal ablation catheter equipped with a CF sensor (ThermoCool SmartTouch SF, Johnson & Johnson MedTech) in combination with a voltage‐controlled PFA generator (biphasic, monopolar 10.65 J per application; TRUPULSE 2, Johnson & Johnson MedTech). Lesion depth increased significantly and nonlinearly with both increasing CF (4–65 g) and the number of delivered pulse trains (12, 18, and 24 burst pulses), ranging from 2.7 to 8.8 mm (*p* < 0.001, Figure 6) [32]. These data show that once electrode–tissue contact is achieved, increasing CF further increases lesion depth. In contrast, when the electrode was positioned only 1–2 mm away from the endocardium (confirmed by intracardiac echocardiography), no lesion formation was observed even with delivery of 24 burst pulses, underscoring the critical importance of electrode–tissue contact [32].

**FIGURE 6:**
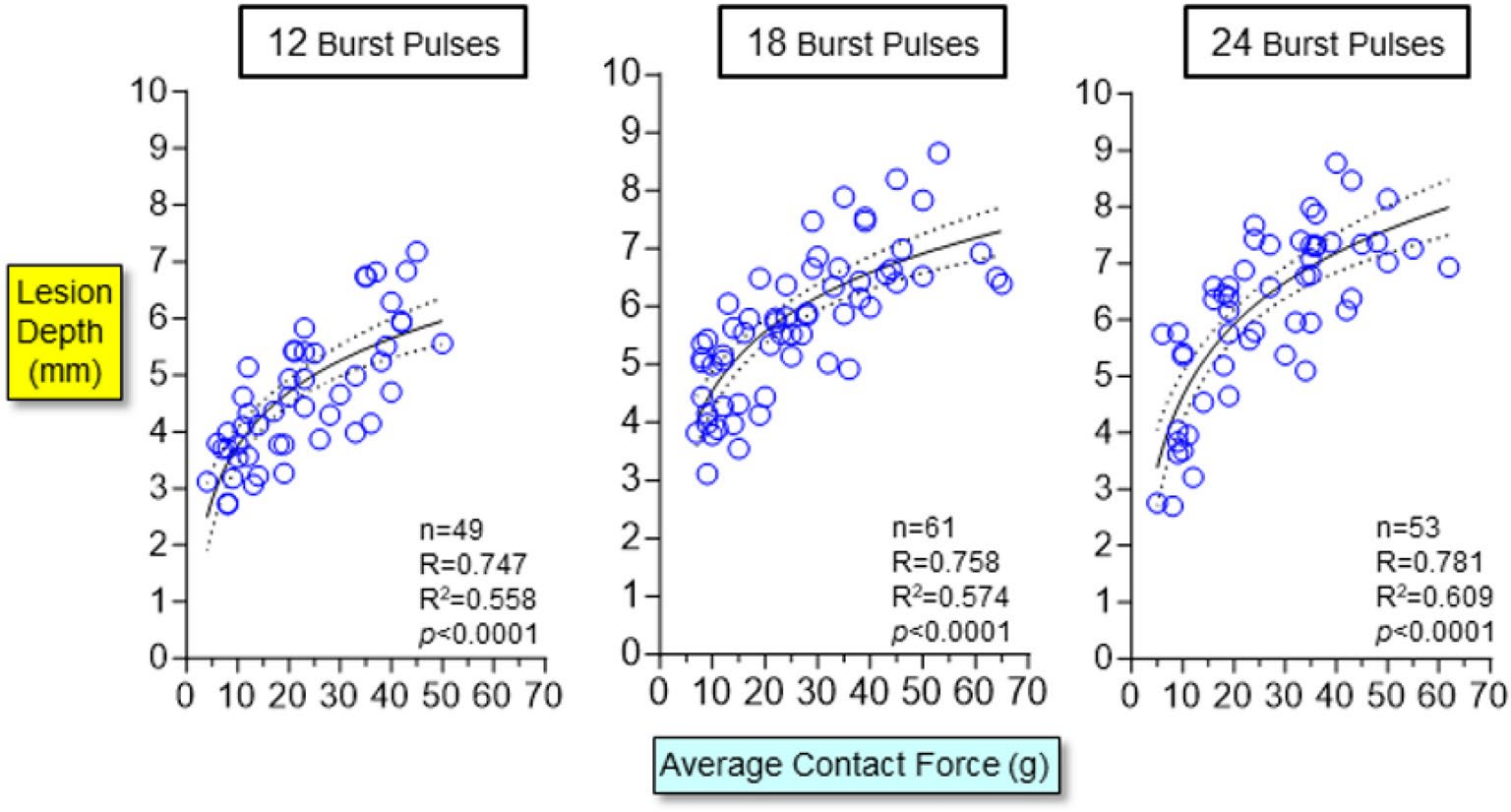
Relationship between average contact force, number of pulsed field ablation (PFA) burst pulses and lesion depth. During 12‐, 18‐, and 24‐burst PFA applications, lesion depth increased significantly with increasing average contact force. From Nakagawa et al. [32] with permission.

Based on these observations, a logarithmic model (**PF Ablation Index**) was developed to predict lesion depth as a function of combined CF and pulse delivery parameters: **PF Ablation Index = *A* × [*B*(*n*) × log(*n*) + *C*(*n*)]**, where *A*, *B*, and *C* are constants, and *n* represents a composite parameter incorporating both the average CF and the number of delivered PFA pulses [31].

Prospective validation of the real‐time predictive accuracy of the PF Ablation Index for lesion depth was performed in a closed‐chest swine model (*n* = 6). PFA was delivered to achieve target lesion depths of 3.5 mm, 4.5 mm, 5.5 mm, and 6.5 mm. The actual measured lesion depth was within ±1.0 mm of the predicted lesion depth in 97 of 106 lesions (92%), demonstrating high predictive accuracy (Figure 7) [31]. Importantly, no lesion formation was observed in the absence of electrode–tissue contact, when the ablation electrode was positioned only 1–2 mm away from the endocardium. In addition, conventional intra‐procedural markers, including intracardiac electrogram attenuation, impedance decrease, and electrode temperature, were poor predictors of PFA lesion size [31].

**FIGURE 7:**
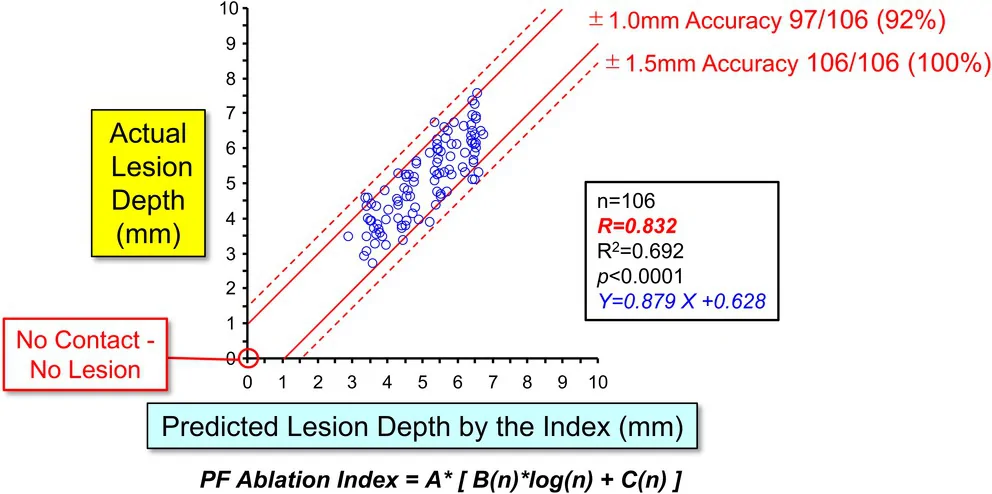
The relationship between the PF Ablation Index‐predicted lesion depth and actual lesion depth. The graph demonstrates accurate prediction of lesion depth, with ±1.0 mm accuracy in 97/106 (92%) lesions and ±1.5 mm accuracy in all 106 (100%) lesions.

Using a multipolar, large footprint focal PFA catheter with a spherical tip (12 mm diameter, 12 electrodes) incorporating a CF sensor at the distal shaft, in combination with bipolar PFA delivery (OMNYPULSE, Johnson & Johnson MedTech), a new PF‐Ablation Index to predict lesion depth was developed. In a prospective validation study using a closed‐chest swine model (*n* = 6), PFA was delivered to achieve target lesion depths of 3.0 mm, 4.5 mm, and 6.0 mm. The actual lesion depth was within ±1.0 mm of the predicted lesion depth in 51 of 73 lesions (70%) and within ±1.5 mm in 67 of 73 lesions (90%), while no lesion formation occurred without electrode–tissue contact (Figure 8) [40].

**FIGURE 8:**
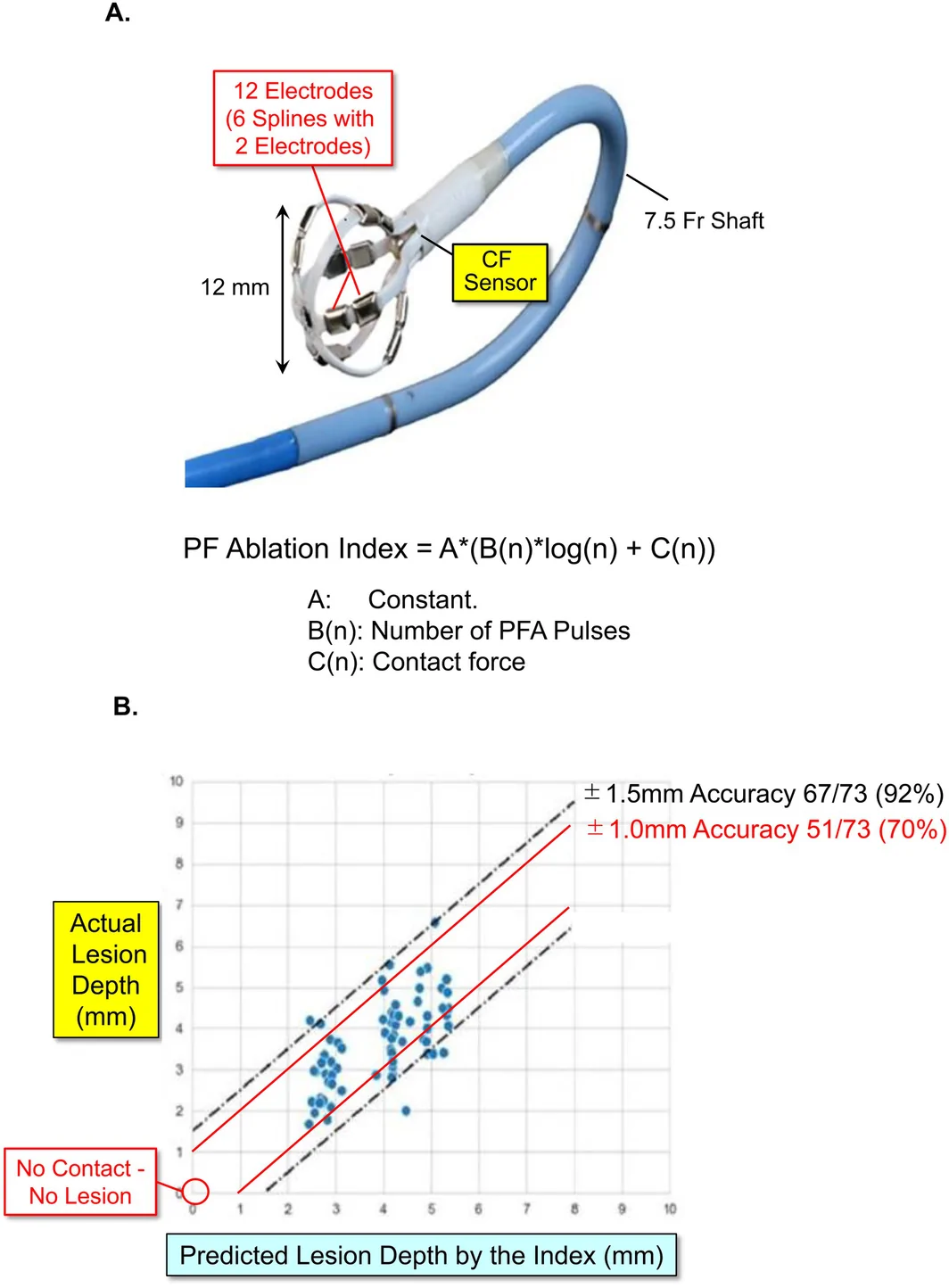
Relationship between the PF Ablation Index‐predicted lesion depth and the actual lesion depth for a multipolar, large footprint focal PFA catheter. (A) A multipolar, large footprint focal PFA catheter with a spherical tip (12‐mm diameter, 12 electrodes) incorporating a contact force sensor equipped at the distal shaft (OMNIPULSE). (B) Relationship between the PF Ablation Index‐predicted lesion depth and the actual lesion depth, showing ±1.0 mm prediction accuracy in 51/73 (70%) lesions and ±1.5 mm accuracy in 67/73 (92%) lesions.

These findings demonstrate that lesion formation with bipolar PFA, similar to monopolar systems, is fundamentally dependent on effective electrode–tissue coupling, with no lesion formation in the absence of electrode contact. However, the slightly lower predictive accuracy of the bipolar PF Ablation Index compared with monopolar systems likely reflects the more complex and spatially heterogeneous electric field distribution inherent to bipolar energy delivery, which is influenced by the number and geometry of actively coupled electrodes. Accordingly, incorporating real‐time assessment of the number of electrodes in tissue contact into prediction models may further improve the accuracy of predicting lesion size with multipolar bipolar PFA systems.

### 1.5 System Heterogeneity in PFA Technology

An important challenge in interpreting the rapidly expanding pre‐clinical and clinical studies on PFA is the substantial heterogeneity among currently available PFA systems. In RF ablation, lesion formation is governed primarily by thermal energy delivery and therefore exhibits relatively predictable characteristics across platforms. In contrast, PFA lesion formation depends on the spatial distribution of electric fields generated by specific waveform parameters and electrode/catheter configurations. As a result, catheter architecture, electrode geometry, energy delivery mode, and waveform design can markedly influence both lesion characteristics and safety profiles.

Several PFA platforms have been developed with distinct design philosophies. Large, multi‐electrode circular, basket, or global electrode catheters have been designed to deliver PFA energy simultaneously across multiple electrodes to facilitate rapid PV isolation, whereas focal catheters allow point‐by‐point energy delivery with greater flexibility in targeting specific myocardial substrates, including linear lesions and deeper ventricular lesion formation [34, 35, 36, 41, 42].

Other systems employ smaller lattice or expandable electrode configurations designed to optimize electric field distribution across the ablation electrode surface [40, 42]. Importantly, systems differ in their use of monopolar versus bipolar energy delivery, pulse voltage/current, pulse duration, pulse sequencing, and waveforms [21, 31, 32, 33, 34, 35, 36, 37, 38, 39, 40, 41, 42, 43]. These parameters strongly influence the delivery of the electric field into the myocardium and blood pool, thereby determining lesion size, lesion geometry, interface or tissue heating, and the susceptibility of adjacent structures (including red blood cells) to electroporation. The heterogeneity of these technologies has important implications when interpreting clinical outcomes and safety observations. Differences in waveform characteristics and catheter design significantly contribute to variability in lesion durability, procedural efficiency, and the incidence of complications, including hemolysis‐related complications, microbubble formation, cerebral embolic events, proarrhythmia, and sudden cardiac death [44, 45, 46, 47, 48, 49]. Consequently, findings reported with one PFA platform should not necessarily be generalized to other systems. A clear understanding of these technological differences is therefore essential when comparing clinical studies and when defining the optimal role of PFA within contemporary catheter ablation strategies.

### 1.6 Safety Considerations in PFA: Hemolysis, Microbubbles, Embolic Risk, Kidney Injury, and Sudden Cardiac Death

Although PFA has been widely promoted as a tissue‐selective and potentially safer alternative to thermal ablation technologies, accumulating experimental and clinical observations suggest that several important safety considerations require careful evaluation.

Ramirez et al., in a review article, reported tissue‐specific thresholds for irreversible electroporation (V/cm) as follows: myocardium, 400; kidney, 600; red blood cells (RBCs), 1600; vascular smooth muscle cells (VSMCs), 1750; endothelium, 1750; and nerve, 3800 [50]. However, as the authors themselves noted, these values were derived from studies employing heterogeneous waveforms and delivery parameters; therefore, a direct comparison of tissue selectivity would be challenging. Based on these estimates, RBCs appear approximately fourfold more resistant to electroporation than myocardium, VSMCs and endothelial cells ~4.4‐fold more resistant, and neural tissue ~9.5‐fold more resistant [50].

Accordingly, reported differences in tissue selectivity should be interpreted with caution, as they may convey a false sense of security regarding the safety of PFA. Clinically, repeated PFA applications after apparent elimination of PV and antral potentials may constitute unnecessary energy delivery and may increase the risk of complications, including acute kidney injury [44, 45, 51, 52]. This concern is supported by accumulating evidence that high‐voltage electric pulse delivery can induce hemolysis, resulting in the release of free hemoglobin and downstream endothelial dysfunction and platelet aggregation [45, 52, 53, 54]. Such effects may contribute to vascular injury and microcirculatory impairment, providing a plausible link to emerging reports of late coronary vasospasm, myocardial ischemia, ventricular fibrillation, and sudden cardiac death following PFA [48, 49, 55, 56, 57, 58, 59, 60].

Another safety consideration relates to the generation of microbubbles during PFA delivery. Historically, during RF ablation with an internally irrigated ablation catheter, microbubble formation associated with overheating at the electrode–tissue interface has been observed [61]. Intracardiac and carotid echocardiography studies have demonstrated microbubble formation during PFA applications, likely reflecting rapid electrochemical reactions, cavitation, and localized changes in dissolved gases near the electrode surface, which may result in nitrogen displacement and hydrolysis [46, 62, 63]. Although higher‐voltage/high‐power PFA delivery is generally associated with increased microbubble generation, waveform optimization during Varipulse applications has been shown to markedly reduce microbubble formation while preserving equivalent lesion formation, despite identical bipolar voltage delivery (1800 V, Videos 1 and 2) [64]. Intracardiac echocardiography during PV isolation using the Farapulse PFA system has demonstrated more frequent and vigorous microbubble generation with the basket configuration than with the flower configuration, likely reflecting increased current density between closely spaced splines during bipolar PFA delivery and possible arcing‐related bubble formation (Video 3).

**VIDEO 1:** PFA applications with Waveform 1 are associated with microbubble formation. See the text for details. Video content can be viewed at https://onlinelibrary.wiley.com/doi/10.1002/joa3.70482.

**VIDEO 2:** Waveform 2 PFA applications demonstrating no increase of microbubble during PFA. See the text for details. Video content can be viewed at https://onlinelibrary.wiley.com/doi/10.1002/joa3.70482.

**VIDEO 3:** Microbubble formation during bipolar PFA delivery. Brisk microbubble generation was observed at the right inferior pulmonary vein ostium during PFA delivery using the Farapulse system in the basket electrode configuration. Abbreviation: RIPV, Right inferior pulmonary vein. See the test for details. Video content can be viewed at https://onlinelibrary.wiley.com/doi/10.1002/joa3.70482.

Although these microbubbles are typically transient, their formation raises concerns regarding the potential for microembolization and cerebral embolic events. Silent cerebral lesions detected by diffusion‐weighted brain MRI have been reported following AF ablation procedures using both thermal (RF and cryo‐thermia) and PFA technologies, suggesting that embolic mechanisms may remain relevant even with PFA modalities [65, 66].

The incidence of silent cerebral events with Varipulse was significantly reduced by increasing the saline irrigation rate from 4 mL/min to 30 mL/min, as evidenced by a decrease in MRI‐detected silent cerebral lesions from 33.3% (6/18 patients) to 4.8% (1/21 patients) [67]. These findings suggest that saline irrigation plays a critical role in modulating PFA‐related complications. Higher irrigation rates may reduce electrode–tissue interface temperature, thereby mitigating heat‐related thrombus formation [64, 68, 69], while also reducing direct PFA current–blood interactions and potentially limiting hemolysis. Collectively, these observations suggest that procedural factors, including irrigation strategy, can substantially influence the embolic and hematologic safety profile of PFA, challenging the assumption that PFA is inherently non‐thrombogenic.

Intravascular hemolysis may trigger a cascade of toxic biological responses through several interrelated mechanisms, including: (1) dissemination of cell‐free hemoglobin (Hb) across tissue barriers; (2) nitric oxide (NO) depletion and Hb‐mediated oxidative stress; (3) release of free heme and iron; and (4) downstream adaptive and pathophysiological processes, ultimately leading to tissue injury involving the kidney, brain, and myocardium [44, 45, 51, 52, 70, 71, 72].

Repeated PFA delivery in patients undergoing AF ablation has been associated with elevated plasma‐free hemoglobin (Hb), hemoglobinuria, increased serum creatinine levels, and acute kidney injury requiring hemodialysis [44, 45]. Recent experimental studies have shown that hemoglobinuria‐related acute kidney injury is driven by intrarenal oxidative reactions and activation of a heme toxicity pathway [51]. Importantly, plasma free‐Hb levels increase significantly with both increasing numbers of PFA applications and inadequate electrode–tissue contact, indicating that unnecessary PFA energy delivery in the absence of electrode‐myocardial contact may substantially increase hemolysis‐related toxicity and should therefore be minimized [72].

Importantly, PFA‐induced intravascular hemolysis appears to be highly dependent on the specific PFA platform and energy‐delivery strategy. In a preclinical comparison, Wright et al. observed markedly higher peak plasma‐free hemoglobin levels with Farawave than with Varipulse or focal Dual Energy ST‐SF PFA (129 vs. 39 vs. 5 mg/dL; *p* < 0.001, respectively), with severe hemolysis (> 50 mg/dL) occurring in all Farawave‐treated animals but no meaningful hemolysis with focal Dual Energy ST‐SF PFA [73].

Phrenic nerve (PN) injury is a recognized complication of thermal ablation, with a reported incidence of 0.3%–0.48% [13, 74]. Although most cases recover spontaneously, persistent PN injury can result in significant long‐term morbidity. Although previously discussed tissue‐selectivity data suggested that the nerve exhibits substantial resistance to electroporation, recent reports have documented both transient and permanent PN paralysis following PFA [75, 76]. The frequent use of paralytic agents to suppress muscle twitching during PF delivery may further obscure intraprocedural PN localization using phrenic stimulation, potentially limiting the ability to recognize impending nerve injury in real time.

As demonstrated in numerous preclinical and clinical studies, endocardial and epicardial PFA delivered in proximity to the coronary arteries frequently induces coronary narrowing [18, 55, 56, 57]. Because intravenous nitroglycerin administration attenuates this narrowing, coronary vasospasm has been considered the primary underlying mechanism [55, 56, 57]. However, clinical cases exhibiting persistent ST‐segment elevation despite nitroglycerin administration have also been reported, suggesting that complete prevention of coronary spasm may not be achievable [60].

Furthermore, histological examination in preclinical swine studies following PFA delivery near coronary arteries has demonstrated segmental necrosis involving the intimal and medial layers, as well as chronic intimal proliferation during follow‐up [18, 58, 77]. These findings raise important concerns about the reliability of previously proposed tissue‐selectivity concepts, suggesting that vascular smooth muscle cells (VSMCs) are substantially more resistant to electroporation than myocardial cells [50].

Importantly, delayed diffuse coronary spasm and ventricular fibrillation have also been reported even in cases in which PFA was not delivered directly adjacent to the coronary arteries [48, 59, 60, 78, 79]. Potential mechanisms may include PFA‐induced hemolysis, leading to NO depletion, endothelial dysfunction, and enhanced platelet aggregation, as well as autonomic instability resulting from stimulation of the ganglionated plexus during pulse delivery. However, further detailed preclinical and clinical investigations are required to clarify the mechanisms underlying these potentially serious vascular and arrhythmic complications.

A multicenter international analysis involving 6721 patients undergoing PFA‐based AF ablation reported 11 life‐threatening delayed or prolonged ischemic and arrhythmic adverse events. These included 5 cases of delayed ST‐segment elevation occurring 4–238 min (median 20 min) after the final PFA delivery, including 1 fatal case associated with subsequent ventricular fibrillation; 2 cases of intraprocedural ventricular fibrillation requiring cardiopulmonary resuscitation; 2 prolonged vagal reactions despite PFA delivery remote from the conduction system (high‐degree atrioventricular block lasting 48 h in 1 patient and prolonged junctional rhythm lasting 240 min in another); and 2 cases of sudden cardiac death occurring 3 and 22 days after the procedure [49].

### 1.7 Future Directions and Unresolved Questions in PFA

Despite the rapid clinical adoption of PFA, several important questions remain unresolved regarding the optimal application of this technology in catheter‐based arrhythmia therapy. First, a more precise understanding of the determinants of irreversible electroporation within the myocardium is needed. The relationships among electric field strength, electrode configuration, waveform characteristics, and tissue properties remain incompletely defined, and these factors likely play a central role in determining lesion size, lesion geometry, and long‐term durability. Improved mechanistic understanding will be essential for optimizing energy‐delivery parameters and catheter designs that produce predictable, durable myocardial lesions.

Second, the role of electrode–tissue contact and tissue geometry in shaping electric field distribution warrants further investigation. Experimental studies suggest that even a minimal separation between the electrode and the myocardial surface can markedly reduce the effective electric field intensity within the tissue. In regions with complex anatomy—such as trabeculated ventricular myocardium or heterogeneous scar tissue—variability in contact and tissue conductivity may contribute to incomplete lesion formation. Using multi‐electrode PFA systems, future technologies that allow accurate real‐time assessment of electric field distribution and durable lesion size may help improve procedural consistency.

Third, important safety questions remain to be addressed. While PFA has demonstrated a favorable safety profile regarding collateral thermal injury, emerging data suggest that intravascular exposure of blood elements to electric fields may result in hemolysis, microbubble formation, and potential embolic phenomena. The biological consequences of these effects, including NO depletion, endothelial dysfunction, and platelet activation, require further mechanistic and clinical investigation. Many currently available PFA systems do not provide a reliable procedural endpoint based solely on electrogram elimination or arrhythmia termination. Consequently, operators may be inclined to deliver unnecessary repeated PFA applications, potentially increasing the risk of serious complications that were rarely encountered with conventional thermal ablation. These complications may represent a distinct spectrum of PFA‐related systemic injury, for which we propose “ALH syndrome” (“Ablate Like Hell” syndrome) as a hypothesis‐generating conceptual framework rather than an established clinical syndrome, including renal, neurological, vascular, and fatal cardiac events. At present, a prudent strategy to mitigate the potential risk of such complications is to minimize unnecessary or excessive PFA applications. In particular, real‐time lesion prediction systems—such as a PF Ablation Index integrating parameters including CF, tissue coupling, and number of PFA pulses—may enable accurate prediction of irreversible lesion size during PFA delivery, thereby minimizing unnecessary energy applications, especially in the absence of electrode–tissue contact. Whether index‐guided PFA strategies can improve the efficacy and clinical outcomes of PFA while reducing the risk of serious PFA‐specific complications remains to be determined. Further evidence is awaited from ongoing clinical studies evaluating these strategies.

Finally, the increasing diversity of PFA platforms highlights the need for standardized approaches to evaluating lesion formation and clinical outcomes. Differences in electrode configuration, waveform characteristics, and energy delivery strategies may lead to substantial variability in lesion durability and safety profiles among systems. Comparative studies and longer‐term clinical follow‐up will be necessary to define best practices and to determine how PFA technologies should be integrated into contemporary ablation strategies.

## 2 Conclusions

Although a comprehensive understanding of the unique biological effects of PFA remains incomplete, vigilant safety surveillance, together with continued refinement of procedural strategies, will be essential for optimizing the safe and effective clinical application of this technology. Ultimately, the long‐term success of PFA will depend not only on its ability to achieve rapid acute electrical isolation but also on the development of mechanistically guided approaches that ensure durable lesion formation while minimizing systemic and collateral tissue injury, preventing “ALH syndrome.”

## Funding

The authors have nothing to report.

## Disclosure

Dr. Nakagawa is a consultant for Johnson & Johnson MedTech Inc., Abbott Inc., Cardiofocus Inc., Stereotaxis Inc., Philips Japan Inc., Japan Lifeline Ltd. and Fukuda Denshi Ltd. Drs. Jackman and Ikeda are consultants for Johnson & Johnson MedTech Inc. Drs. Farshchi‐Heydari and Maffre are employees of Johnson & Johnson MedTech Inc. Dr. Castellvi is an employee of CardioFocus Inc.

## Conflicts of Interest

The authors declare no conflicts of interest.

## Data Availability

The data that support the findings of this study are available from the corresponding author upon reasonable request.

## References

[1] J. Verdell , R. Frank , G. Fontaine , J. F. Fournial , and Y. Grosgogeat , “Permanent Intra‐Hisian Atrioventricular Block Induced During Right Intraventricular Exploration,” Archives des Maladies du Coeur et des Vaisseaux 72, no. 1 (1979): 107–112. 107875

[2] R. Gonzalez , M. Scheinman , W. Margaretten , and M. Rubinstein , “Closed‐Chest Electrode‐Catheter Technique for His Bundle Ablation in Dogs,” American Journal of Physiology 241, no. 2 (1981): H283–H287. 10.1152/ajpheart.1981.241.2.H283 7270717

[3] M. M. Scheinman , F. Morady , D. S. Hess , and R. Gonzalez , “Catheter‐Induced Ablation of the Atrioventricular Junction to Control Refractory Supraventricular Arrhythmias,” JAMA 248, no. 7 (1982): 851–855. 7097946

[4] J. J. Gallagher , R. H. Svenson , J. H. Kasell , et al., “Catheter Technique for Closed‐Chest Ablation of the Atrioventricular Conduction System,” New England Journal of Medicine 306, no. 4 (1982): 194–200. 10.1056/NEJM198201283060402 7054682

[5] B. B. Lerman , J. L. Weiss , B. H. Bulkley , L. C. Becker , and M. L. Weisfeldt , “Myocardial Injury and Induction of Arrhythmia by Direct Current Shock Delivered via Endocardial Catheters in Dogs,” Circulation 69, no. 5 (1984): 1006–1012. 10.1161/01.cir.69.5.1006 6705156

[6] H. Nakagawa , M. Okamoto , K. Nagata , et al., “Efficacy and Safety of Catheter Electrical Ablation: Experimental and Clinical Studies,” Circulation Journal 54, no. 10 (1990): 1356–1364. 10.1253/jcj.54.10_1356 2277415

[7] G. T. Evans, Jr. , M. M. Scheinman , D. P. Zipes , et al., “Catheter Ablation for Control of Ventricular Tachycardia: A Report of the Percutaneous Cardiac Mapping and Ablation Registry,” Pacing and Clinical Electrophysiology 9, no. 6 Pt. 2 (1986): 1391–1395. 10.1111/j.1540-8159.1986.tb06729.x 2432568

[8] R. Lemery , M. Talajic , D. Roy , et al., “Success, Safety, and Late Electrophysiological Outcome of Low‐Energy Direct‐Current Ablation in Patients With the Wolff‐Parkinson‐White Syndrome,” Circulation 85, no. 3 (1992): 957–962. 10.1161/01.cir.85.3.957 1537132

[9] A. J. Ahsan , D. Cunningham , E. Rowland , and A. F. Rickards , “Catheter Ablation Without Fulguration: Design and Performance of a New System,” Pacing and Clinical Electrophysiology 12, no. 9 (1989): 1557–1561, 10.1111/j.1540-8159. 2476786

[10] M. Borggrefe , T. Budde , A. Podczeck , and G. Breithardt , “High Frequency Alternating Current Ablation of an Accessory Pathway in Humans,” Journal of the American College of Cardiology 10, no. 3 (1987): 576–582. 10.1016/s0735-1097(87)80200-0 3624664

[11] W. M. Jackman , X. Wang , K. J. Friday , et al., “Catheter Ablation of Accessory Atrioventricular Pathways (Wolff‐Parkinson‐White Syndrome) by Radiofrequency Current,” New England Journal of Medicine 324, no. 23 (1991): 1605–1611. 10.1056/NEJM199106063242301 2030716

[12] H. Calkins , J. Sousa , S. Rosenheck , et al., “Diagnosis and Cure of the Wolff‐Parkinson‐White Syndrome or Paroxysmal Supraventricular Tachycardias During a Single Electrophysiologic Test,” New England Journal of Medicine 324, no. 23 (1991): 1612–1618. 10.1056/NEJM199106063242302 2030717

[13] K. H. Kuck , J. Josep Brugada , A. Furnkranz , et al., “Cryoballoon or Radiofrequency Ablation for Paroxysmal Atrial Fibrillation,” New England Journal of Medicine 374, no. 23 (2016): 2235–2245, 10.1056/NEJMoa1602014. 27042964

[14] H. Nakagawa , A. Ikeda , T. Sharma , et al., “Comparison of in Vivo Tissue Temperature Profile and Lesion Geometry for Radiofrequency Ablation With High Power‐Short Duration and Moderate Power‐Moderate Duration: Effects of Thermal Latency and Contact Force on Lesion Formation,” Circulation. Arrhythmia and Electrophysiology 14, no. 7 (2021): e009899, 10.1161/CIRCEP.121.009899. 34138641

[15] M. E. Haddad , P. Taghji , T. Phlips , et al., “Determinants of Acute and Late Pulmonary Vein Reconnection in Contact Force–Guided Pulmonary Vein Isolation Identifying the Weakest Link in the Ablation Chain,” Circulation: Arrhythmia and Electrophysiology 10, no. 4 (2017): e004867, 10.1161/CIRCEP.116.004867. 28381417

[16] J. Lavee , G. Onik , P. Mikus , and B. Rubinsky , “A Novel Nonthermal Energy Source for Surgical Epicardial Atrial Ablation: Irreversible Electroporation,” Heart Surgery Forum 10, no. 2 (2007): E162–E167, 10.1532/HSF98.20061202. 17597044

[17] H. Nakagawa and W. M. Jackman , “Electroporation (Revival of Direct Current Ablation): New Approach for Increasing Epicardial Ablation Safety in Close Proximity to a Coronary Artery,” Circulation. Arrhythmia and Electrophysiology 7, no. 5 (2014): 779–780. 10.1161/CIRCEP.114.002255 25336363

[18] K. Neven , F. Wittkampf , V. van Driel , et al., “Safety and Feasibility of Closed Chest Epicardial Catheter Ablation Using Electroporation,” Circulation: Arrhythmia and Electrophysiology 7, no. 5 (2014): 913–919. 10.1161/CIRCEP.114.001607 25156260

[19] E. Neumann , M. Schaefer‐Ridder , Y. Wang , and P. H. Hofschneider , “Gene Transfer Into Mouse Lyoma Cells by Electroporation in High Electric Fields,” EMBO Journal 1, no. 7 (1982): 841–845. 10.1002/j.1460-2075.1982.tb01257.x PMC553119 6329708

[20] J. Hong , M. T. Stewart , D. S. Cheek , D. E. Francischelli , and N. Kirchhof , “Cardiac ablation via electroporation,” Annual International Conference of the IEEE Engineering in Medicine and Biology Society 209 (2009): 3381–3384, 10.1109/IEMBS.2009.5332816. 19963798

[21] H. Nakagawa , Q. Castellvi , R. Neal , et al., “Effects of Contact Force on Lesion Size During Pulsed Field Catheter Ablation: Histochemical Characterization of Ventricular Lesion Boundaries,” Circulation. Arrhythmia and Electrophysiology 17, no. 1 (2024): e012026. 10.1161/CIRCEP.123.012026 38152949

[22] M. Matsuda , H. Fujiwara , T. Onodera , et al., “Quantitative Analysis of Infarct Size, Contraction Band Necrosis, and Coagulation Necrosis in Human Autopsied Hearts With Acute Myocardial Infarction After Treatment With Selective Intracoronary Thrombolysis,” Circulation 76, no. 5 (1987): 981–989, 10.1161/01.cir.76.5.981. 3665003

[23] S. Corovic , B. Markelc , M. Dolinar , M. Cemazar , and T. Jarm , “Modeling of Microvascular Permeability Changes After Electroporation,” PLoS One 10, no. 3 (2015): e0121370, 10.1371/journal.pone.0121370. PMC4368817 25793292

[24] W. C. Stoffregen , S. D. Rousselle , and M. K. Rippy , “Pathology Approaches to Determine Safety and Efficacy of Cardiac Ablation Catheters,” Toxicologic Pathology 47, no. 3 (2019): 311–328, 10.1177/0192623319826063. 30727858

[25] N. T. Batista , T. Polajzer , and D. Miklavcic , “Cell Death due to Electroporation ‐ a Review,” Bioelectrochemistry 141 (2021): 107871, 10.1016/j.bioelechem.2021.107871. 34147013

[26] E. Maor , A. Sugrue , C. Witt , et al., “Pulsed Electric Fields for Cardiac Ablation and Beyond: A State‐Of‐The‐Art Review,” Heart Rhythm 16, no. 7 (2019): 1112–1120, 10.1016/j.hrthm.2019.01.01. 30641148

[27] M. Faroja , M. Ahmed , L. Appelbaum , et al., “Irreversible Electroporation Ablation: Is All the Damage Nonthermal?,” Radiology 266, no. 2 (2013): 462–470, 10.1148/radiol.12120609. 23169795

[28] S. Jeong , H. Kim , J. Park , K. W. Kim , S. B. Sim , and J. H. Chung , “Evaluation of Electroporated Area Using 2,3,5‐Triphenyltetrazolium Chloride in a Potato Model,” Scientific Reports 11 (2021): 20431, 10.1038/s41598-021-99987-2. PMC8516888 34650212

[29] E. L. Hansen , E. B. Sozer , S. Romeo , S. K. Frandsen , P. T. Vernier , and J. Gehl , “Dose‐Dependent ATP Depletion and Cancer Cell Death Following Calcium Electroporation, Relative Effect of Calcium Concentration and Electric Field Strength,” PLoS One 10, no. 4 (2015): e0122973, 10.1371/journal.pone.0122973. PMC4390219 25853661

[30] J. M. Ross , “Visualization of Mitochondrial Respiratory Function Using Cytochrome c Oxidase/Succinate Dehydrogenase (COX/SDH) Double‐Labeling Histochemistry,” Journal of Visualized Experiments 30, no. 57 (2011): e3266, 10.3791/3266. PMC3308593 22143245

[31] H. Nakagawa , S. Farshchi‐Heydari , M. Sugawara , et al., “Real‐Time Prediction of Irreversible Lesion Size During Pulsed Field Ablation: Prospective Validation of a Novel Ablation Index Based on Contact Force and Number of Applications in a Swine Beating Heart Model,” Circulation. Arrhythmia and Electrophysiology 18, no. 12 (2025): e013911, 10.1161/CIRCEP.125.013911. PMC12711265 41328568

[32] H. Nakagawa , S. Farshchi‐Heydari , J. Maffre , et al., “Evaluation of Ablation Parameters to Predict Irreversible Lesion Size During Pulsed Field Ablation,” Circulation. Arrhythmia and Electrophysiology 17, no. 8 (2024): e012814, 10.1161/CIRCEP.124.012814. 39051118

[33] A. Verma , D. E. Haines , L. V. Boersma , et al., “Pulsed Field Ablation for the Treatment of Atrial Fibrillation: PULSED AF Pivotal Trial,” Circulation 147, no. 19 (2023): 1422–1432, 10.1161/CIRCULATIONAHA.123.063988. PMC10158608 36877118

[34] M. Duytschaever , T. D. Potter , M. Grimaldi , et al., “Paroxysmal Atrial Fibrillation Ablation Using a Novel Variable‐Loop Biphasic Pulsed Field Ablation Catheter Integrated With a 3‐Dimensional Mapping System: 1‐Year Outcomes of the Multicenter inspIRE Study,” Circulation. Arrhythmia and Electrophysiology 16 (2023): e011780, 10.1161/CIRCEP.122.011780. PMC10026968 36735937

[35] V. Y. Reddy , E. P. Gerstenfeld , A. Natale , et al., “Pulsed Field or Conventional Thermal Ablation for Paroxysmal Atrial Fibrillation,” New England Journal of Medicine 389, no. 18 (2023): 1660–1671. 10.1056/NEJMoa2307291 37634148

[36] T. Kueffer , S. Bordignon , K. Kars Neven , et al., “Durability of Pulmonary Vein Isolation Using Pulsed‐Field Ablation. Results From the Multicenter EU‐PORIA Registry,” JACC: Clinical Electrophysiology 10, no. 4 (2024): 698–708, 10.1016/j.jacep.2023.11.026. 38340118

[37] A. M. Amin , A. Nazir , M. T. Abuelazm , et al., “Efficacy and Safety of Pulsed‐Field Versus Conventional Thermal Ablation for Atrial Fibrillation: A Systematic Review and Meta‐Analysis,” Journal of Arrhythmia 40, no. 5 (2024): 1059–1074. 10.1002/joa3.13118 PMC11474578 39416254

[38] I. Martins Esteves , V. B. de Pontes , T. Trevisan , S. Jaramillo , and W. F. Gomes , “Pulsed‐Field Ablation Versus Radiofrequency Ablation for Paroxysmal Atrial Fibrillation: A Systematic Review and Meta‐Analysis,” Journal of Arrhythmia 41, no. 4 (2025): e70158, 10.1002/joa3.70158. PMC12320123 40761535

[39] A. Verma , R. Neal , J. Evans , et al., “Characteristics of Pulsed Electric Field Cardiac Ablation Porcine Treatment Zones With a Focal Catheter,” Journal of Cardiovascular Electrophysiology 34, no. 1 (2023): 99–107, 10.1111/jce.15734. PMC10100505 36335638

[40] L. Di Biase , J. Marazzato , A. Govari , et al., “Pulsed Field Ablation Index–Guided Ablation for Lesion Formation: Impact of Contact Force and Number of Applications in the Ventricular Model,” Circulation. Arrhythmia and Electrophysiology 17, no. 4 (2024): e012717, 10.1161/CIRCEP.123.012717. PMC11017832 38390748

[41] M. K. Turagam , P. Neuzil , B. Boris Schmidt , et al., “Safety and Effectiveness of Pulsed Field Ablation to Treat Atrial Fibrillation: One‐Year Outcomes From the MANIFEST‐PF Registry,” Circulation 148, no. 1 (2023): 35–46, 10.1161/CIRCULATIONAHA.123.064959. 37199171

[42] V. Y. Reddy , H. Kottkamp , H. Calkins , et al., “Pulsed‐Field Ablation to Enhance the Durability of Pulmonary Vein Isolation and Treat Paroxysmal Atrial Fibrillation,” Journal of the American College of Cardiology 87, no. 14 (2026): 1747–1761, 10.1016/j.jacc.2025.10.084. 41563170

[43] V. Y. Reddy , P. Peichl , E. Elad Anter , et al., “A Focal Ablation Catheter Toggling Between Radiofrequency and Pulsed Field Energy to Treat Atrial Fibrillation,” JACC: Clinical Electrophysiology 9, no. 8 Pt. 3 (2023): 1786–1801, 10.1016/j.jacep.2023.04.002. 37227340

[44] S. Venier , N. Vaxelaire , P. Jacon , et al., “Severe Acute Kidney Injury Related to Haemolysis After Pulsed Field Ablation for Atrial Fibrillation,” Europace 26, no. 1 (2024): euad371, 10.1093/europace/euad371. PMC10776308 38175788

[45] S. Mohanty , M. Casella , P. Compagnucc , et al., “Acute Kidney Injury Resulting From Hemoglobinuria After Pulsed‐Field Ablation in Atrial Fibrillation. Is It Preventable?,” JACC: Clinical Electrophysiology 10, no. 4 (2024): 709–715, 10.1016/j.jacep.2023.12.008. 38310489

[46] S. Shiomi , M. Tokuda , U. Takato , et al., “Real‐Time Detection of Microembolic Signals Using Carotid Echocardiography: A Comparative Study of 3 Pulsed‐Field Systems,” Heart Rhythm 23, no. 1 (2026): 25–31. 10.1016/j.hrthm.2025.06.046 40736427

[47] S. Miyazaki , I. Kawamura , Y. Iwasa , et al., “Different Incidence and Size of Silent Strokes After Pulsed Field Ablation With Circular Shaped Ablation Catheters,” Circulation. Arrhythmia and Electrophysiology 18, no. 5 (2025): e013719, 10.1161/CIRCEP.125.013719. 40270255

[48] G. Matsuura , H. Fukaya , T. Yoshizawa , A. Sugimoto , and J. Ako , “Delayed Coronary Artery Vasospasm Leading to Cardiac Arrest After Pulsed Field Ablation for Atrial Fibrillation,” Heart Rhythm 23, no. 2 (2026): e287–e289, 10.1016/j.hrthm.2025.10.010. 41072746

[49] M. Gunawardene , B. Schmidt , P. Peichl , et al., “Life‐Threatening Delayed Myocardial Ischemia and Malignant Arrhythmias Occurring After Pulsed Field Ablation of Atrial Fibrillation,” Circulation 153, no. 10 (2026): 789–793, 10.1161/CIRCULATIONAHA.125.077983. 41446933

[50] F. D. Ramirez , V. Y. Reddy , R. Viswanathan , M. Hocini , and P. Jaïs , “Emerging Technologies for Pulmonary Vein Isolation,” Circulation Research 127, no. 1 (2020): 170–183, 10.1161/CIRCRESAHA.120.316402. 32716722

[51] J. W. Deuel , C. A. Schaer , F. S. Boretti , et al., “Hemoglobinuria‐Related Acute Kidney Injury Is Driven by Intrarenal Oxidative Reactions Triggering a Heme Toxicity Response,” Cell Death and Disease 7, no. 1 (2016): e2064, 10.1038/cddis.2015.392. PMC4816175 26794659

[52] P. W. Buehler , R. Humar , and D. J. Schaer , “Haptoglobin Therapeutics and Compartmentalization of Cell‐Free Hemoglobin Toxicity,” Trends in Molecular Medicine 26, no. 7 (2020): 683–697, 10.1016/j.molmed.2020.02.004. 32589936

[53] Y. Higashi , “Noninvasive Assessment of Vascular Function: From Physiological Test to Biomarkers,” JACC Asia 4, no. 12 (2024): 898–911, 10.1016/j.jacasi.2024.09.015. PMC11711812 39802992

[54] C. Gianni , A. Al‐Ahmad , M. Elchouemi , et al., “Pulsed Field Ablation–Related Hemolysis: Comparison Between Technologies,” Circulation. Arrhythmia and Electrophysiology 18, no. 12 (2025): e014021, 10.1161/CIRCEP.125.014021. PMC12711278 41328578

[55] V. Y. Reddy , J. Petru , M. Funasako , et al., “Coronary Arterial Spasm During Pulsed Field Ablation to Treat Atrial Fibrillation,” Circulation 146, no. 24 (2022): 1808–1819. 10.1161/CIRCULATIONAHA.122.061497 36134574

[56] Y. Malyshev , P. Neuzil , J. Petru , et al., “Nitroglycerin to Ameliorate Coronary Artery Spasm During Focal Pulsed‐Field Ablation for Atrial Fibrillation,” JACC Clinical Electrophysiology 10, no. 5 (2024): 885–896, 10.1016/j.jacep.2023.12.015. 38385916

[57] C. Zhang , P. Neuzil , J. Jan Petru , et al., “Coronary Artery Spasm During Pulsed Field vs Radiofrequency Catheter Ablation of the Mitral Isthmus,” JAMA Cardiology 9, no. 1 (2024): 72–77, 10.1001/jamacardio.2023.4405. PMC10687713 38019505

[58] G. Amorós‐Figueras , Q. Quim Castellví , S. Casabella‐Ramon , et al., “Angiographic and Histological Characterization of Pulsed Field Ablation‐Induced Coronary Spasm: Differential Effect of 2 Waveforms,” Heart Rhythm 23, no. 7 (2026): e1186–e1195, 10.1016/j.hrthm.2026.03.1904. 41871647

[59] V. Luther , J. Chiong , C. James , S. Modi , D. Gupta , and J. Hung , “Diffuse Right Coronary Artery Spasm Occurring 45 Minutes After Pulsed Field Ablation for Atrial Fibrillation,” Heart Rhythm 22, no. 7 (2025): 1864–1867. 10.1016/j.hrthm.2025.03.1978 40147721

[60] M. Gunawardene , J. Hartmann , E. Tigges , J. Jezuit , and S. Willems , “Word of Caution: Clinically Apparent Coronary Spasm Following Pulsed Field Cavotricuspid Isthmus Ablation Despite Nitroglycerin Prophylaxis: A Case Report,” European Heart Journal ‐ Case Reports 8, no. 11 (2024): ytae553, 10.1093/ehjcr/ytae553. PMC11561576 39545158

[61] N. F. Marrouche , O. David , D. O. Martin , et al., “Phased‐Array Intracardiac Echocardiography Monitoring During Pulmonary Vein Isolation in Patients With Atrial Fibrillation. Impact on Outcome and Complications,” Circulation 107, no. 21 (2003): 2710–2716. 10.1161/01.CIR.0000070541.83326.15 12756153

[62] M. Ashokkumar , “The Characterization of Acoustic Cavitation Bubbles – An Overview,” Ultrasonics Sonochemistry 18, no. 4 (2011): 864–872, 10.1016/j.ultsonch.2010.11.016. 21172736

[63] S. Mahniˇc‐Kalamiza , D. Miklavcic , P. Lombergar , et al., “Elucidating the Mechanisms of Microbubble Formation in Intracardiac Pulsed Field Ablation,” Electrochimica Acta 497 (2024): 144550, 10.1016/j.electacta.2024.144550.

[64] A. Ikeda , H. Nakagawa , M. Sugawara , et al., “Effects of Waveform and Irrigation Flow Rates on the Electrode–Tissue Interface Heating and Lesion Size During Pulsed‐ Field Ablation in the Swine Thigh Muscle Preparation,” Heart Rhythm 23, no. 4 (2026): S113, 10.1016/j.hrthm.2026.03.342.

[65] T. Deneke , P. Jais , M. Scaglione , et al., “Silent Cerebral Events/Lesions Related to Atrial Fibrillation Ablation: A Clinical Review,” Journal of Cardiovascular Electrophysiology 26, no. 4 (2015): 455–463. 10.1111/jce.12608 25556518

[66] B. Schmidt , M. Gunawardene , D. Krieg , et al., “A Prospective Randomized Single‐Center Study on the Risk of Asymptomatic Cerebral Lesions Comparing Irrigated Radiofrequency Current Ablation With the Cryoballoon and the Laser Balloon,” Journal of Cardiovascular Electrophysiology 24, no. 8 (2013): 869–874. 10.1111/jce.12151 23601001

[67] S. Nakahara , H. Aoki , H. Sato , et al., “Irrigation Flow Rate and Silent Cerebral Events During Pulsed Field Ablation With a Variable‐Loop Catheter,” JACC: Clinical Electrophysiology 12, no. 1 (2026): 165–167. 10.1016/j.jacep.2025.09.007 41369638

[68] W. H. Sauer , D. Campos‐Villarreal , and N. A. Steiger , “Irrigation of Pulsed Field Ablation Electrodes Mitigates Joule Heating and the Heat Stacking Phenomena,” JACC Clinical Electrophysiology 11, no. 8 (2025): 1866–1868. 10.1016/j.jacep.2025.04.018 40392664

[69] E. Zito , M. Mansour , V. Y. Reddy , et al., “Assessment of Temperature Dynamics in Pulsed Field Ablation With a Variable‐Loop Circular Catheter: A Comparative Analysis of Waveform Configurations and Irrigation Rates in Specimens of Bovine Ventricular Myocardium,” Europace 27, no. 11 (2025): euaf278, 10.1093/europace/euaf278. PMC12629080 41166649

[70] R. Rother , L. Bell , P. Hillmen , and M. T. Gladwin , “The Clinical Sequelae of Intravascular Hemolysis and Extracellular Plasma Hemoglobin. A Novel Mechanism of Human Disease,” Journal of the American Medical Association 293, no. 13 (2005): 1653–1662. 10.1001/jama.293.13.1653 15811985

[71] M. A. Popa , S. Venier , R. Menè , et al., “Characterization and Clinical Significance of Hemolysis After Pulsed Field Ablation for Atrial Fibrillation: Results of a Multicenter Analysis,” Circulation. Arrhythmia and Electrophysiology 17, no. 10 (2024): e012732, 10.1161/CIRCEP.124.012732. 39212069

[72] M. Shinohara , C. Oates , J. R. Power , et al., “A Comparative Analysis of Hemolysis Related to the Bipolar Pentaspline and Monopolar Lattice‐Tip Catheters,” Journal of the American College of Cardiology Electrophysiology 12, no. 2 (2025): 387–389, 10.1016/j.jacep.2025.09.039. 41186536

[73] L. E. Wright , M. S. Miller , N. Raissi‐Dehkordi , et al., “Pre‐Clinical Longitudinal Evaluation of Intravascular Hemolysis With Various Pulsed Field Ablation Systems,” Heart Rhythm 23, no. 4 Suppl (2026): S671, 10.1016/j.hrthm.2026.03.1103.

[74] F. Sacher , K. Monahan , S. P. Thomas , et al., “Phrenic Nerve Injury After Atrial Fibrillation Catheter Ablation: Characterization and Outcome in a Multicenter Study,” Journal of the American College of Cardiology 47, no. 12 (2006): 2498–2503. 10.1016/j.jacc.2006.02.050 16781380

[75] F. Pansera , S. Bordignon , F. Bologna , et al., “Catheter Ablation Induced Phrenic Nerve Palsy by Pulsed Field Ablation—Completely Impossible? A Case Series,” European Heart Journal ‐ Case Reports 6, no. 9 (2022): ytac361. 10.1093/ehjcr/ytac361 PMC9477201 36128440

[76] Y. Oda , A. Akihiko Nogami , D. Daisuke Igarashi , et al., “A Case Report of Persistent Phrenic Nerve Injury by Pulsed Field Ablation: Lessons From Anatomical Reconstruction and Electrode Positioning,” Heart Rhythm Case Report 11, no. 8 (2025): 763–766. 10.1016/j.hrcr.2025.05.015 PMC12399187 40896670

[77] S. Higuchi , S. I. I. Im , C. Stillson , et al., “Effect of Epicardial Pulsed Field Ablation Directly on Coronary Arteries,” JACC: Clinical Electrophysiology 8, no. 12 (2022): 1486–1496, 10.1016/j.jacep.2022.09.003. 36779624

[78] Y. Nakamura , T. Matsuyama , T. Ishikawa , et al., “Histopathology of the Atrial Tissue After Pulsed‐Field Ablation: A Subacute‐Phase Lesion in an Autopsy Heart,” Heart Rhythm 23, no. 8 (2026): e1693–e1694, 10.1016/j.hrthm.2026.05.054. 41740822

[79] Y. Okubo , S. Ishida , H. Shimajiri , et al., “Electrical Storm After Pulsed Field Ablation of Atrial Fibrillation in Patients With Impaired Left Ventricular Function,” Heart Rhythm 23 (2026): e287–e289, 10.1016/j.hrthm.2026.02. 42250895

